# EIN3 and SOS2 synergistically modulate plant salt tolerance

**DOI:** 10.1038/srep44637

**Published:** 2017-03-16

**Authors:** Ruidang Quan, Juan Wang, Dexin Yang, Haiwen Zhang, Zhijin Zhang, Rongfeng Huang

**Affiliations:** 1 Biotechnology Research Institute, Chinese Academy of Agricultural Sciences, 12 Zhongguancun South Road, Beijing, 100081, China; 2 National Key Facility of Crop Gene Resources and Genetic Improvement, 12 Zhongguancun South Road, Beijing, 100081, China

## Abstract

Ethylene biosynthesis and the ethylene signaling pathway regulate plant salt tolerance by activating the expression of downstream target genes such as those related to ROS and Na^+^/K^+^ homeostasis. The Salt Overly Sensitive (SOS) pathway regulates Na^+^/K^+^ homeostasis in *Arabidopsis* under salt stress. However, the connection between these two pathways is unclear. Through genetic screening, we identified two *sos2* alleles as salt sensitive mutants in the *ein3-1* background. Neither Ethylene-Insensitive 2 (EIN2) nor EIN3 changed the expression patterns of *SOS* genes including *SOS1, SOS2, SOS3* and *SOS3-like Calcium Binding Protein 8 (SCaBP8*), but SOS2 activated the expression of one target gene of EIN3, *Ethylene and Salt-inducible ERF 1 (ESE1*). Moreover, Ser/Thr protein kinase SOS2 phosphorylated EIN3 *in vitro* mainly at the S325 site and weakly at the S35, T42 and S606 sites. EIN3 S325A mutation reduced its transcriptional activating activity on *ESE1* promoter:GUS in a transient GUS assay, and impaired its ability to rescue *ein3-1* salt hypersensitivity. Furthermore, SOS2 activated salt-responsive ESE1 target gene expression under salt stress. Therefore, EIN3-SOS2 might link the ethylene signaling pathway and the SOS pathway in *Arabidopsis* salt responses.

Salt stress is one of the major factors reducing crop yield. Plant salt stress response pathways consist of ionic and osmotic homeostasis signaling pathways, detoxification response pathways, and pathways for growth regulation^1^,^2^,^3^. Plant hormone signaling, ROS production, transcription regulation, and ionic homeostasis are interconnected and constitute a complex network modulating plant growth and survival under high salt stress^2^,^4^,^5^.

Ethylene is one of the hormones regulating plant development and stress responses, and both ethylene biosynthesis and ethylene signaling affect plant salt responses^6^. The enzyme 1-aminocyclopropane-1-carboxylate synthase (ACS) is crucial in ethylene biosynthesis^7^. Moreover, *ethylene-overproducer 1 (eto1*), an *Arabidopsis* mutation, which negatively regulates ACS activity and ethylene production, is salt tolerant^8^. In ethylene signaling, ethylene is perceived by receptor proteins, Ethylene Response 1/2 (ETR1/2), Ethylene Response Sensor 1/2 (ERS1/2) and Ethylene-INsensitive 4 (EIN4), which physically interact with the kinase Constitutive Triple Response 1 (CTR1) and the metal transporter-like protein EIN2^9^. Ethylene binding leads to CTR1 inactivation, EIN2 dephosphorylation and proteolytic cleavage. Subsequently, the split EIN2 C-terminal fragment is transported into the nucleus and stabilizes EIN3, thus inducing the transcription of *Ethylene-Response-Factor 1 (ERF1*) and other ethylene-responsive genes^10^,^11^,^12^. Constitutive activation of ethylene signaling in *ctr1* leads to salt tolerance, whereas the inactivation of ethylene signaling in *ein2* and *ein3* results in salt sensitivity in *Arabidopsis*^4^,^13^,^14^. The overexpression of either *EIN3* or *Ethylene and Salt-inducible ERF 1 (ESE1*) enhances the expression of salt-responsive genes and salt tolerance in *Arabidopsis*^15^.

Ionic homeostasis in *Arabidopsis* is regulated by the Salt Overly Sensitive (SOS) pathway, consisting of SOS1^16^, SOS2^17^, SOS3^18^ and SOS3-like Calcium Binding Protein 8 (SCaBP8)^19^,^20^. Under salt stress, the calcium binding protein SOS3 senses the increase in cytosolic calcium concentration and then the SOS3-SOS2 protein kinase complex activates the SOS1 ion transporter, which pumps excess cytosolic sodium out of plant cells^1^,^21^,^22^. *SCaBP8*, an *SOS3* homolog, is primarily expressed in shoots, but *SOS3* is primarily expressed in roots^19^.

Crosstalk might exist between different salt response pathways^4^,^23^,^24^. SOS signaling is mainly considered to be sodium homeostasis regulation under salt stress, but the transcription of several hundred genes, including *ERF* genes, changes under salt stress in *sos2* and *sos3* mutants^25^. Ethylene biosynthesis and the ethylene signaling pathway participate in salt stress signaling^6^. Furthermore, ethylene signaling and gibberellin signaling coordinately regulate plant survival and growth under salt stress^14^. Although the mechanism by which both ethylene signaling and SOS pathways regulating plant salt tolerance has been discovered, the connection between ethylene signaling and SOS pathway in salt stress responses remains unknown.

In this study, we isolated two new *sos2* alleles through screening for salt sensitive mutants in the *ein3-1* background and found that SOS2 activates the expression of *ESE1*, which encodes an ERF transcription factor, possibly by phosphorylation and activation of EIN3.

## Results

### EIN3 and SOS2 together regulate salt tolerance in *Arabidopsis*

To isolate salt sensitive mutants in the *ein3-1* background, we constructed an *Arabidopsis* mutation pool by ethyl methane sulfonate (EMS)-mutagenesis of *ein3-1*. And from salt tolerance screening in the M2 seedlings of mutagenized *ein3-1*, we found that two seedlings, 453 and 751, were more sensitive to salt stress than *ein3-1* (Supplementary Fig. S1). A detailed analysis of 453 and 751 mutants using M3 seeds showed that the two mutants had similar salt-sensitive phenotypes (Supplementary Fig. S2). Through map-based cloning, we found that the 453 and 751 mutations were two new alleles of *sos2* mutants^17^.

**Figure 1:**
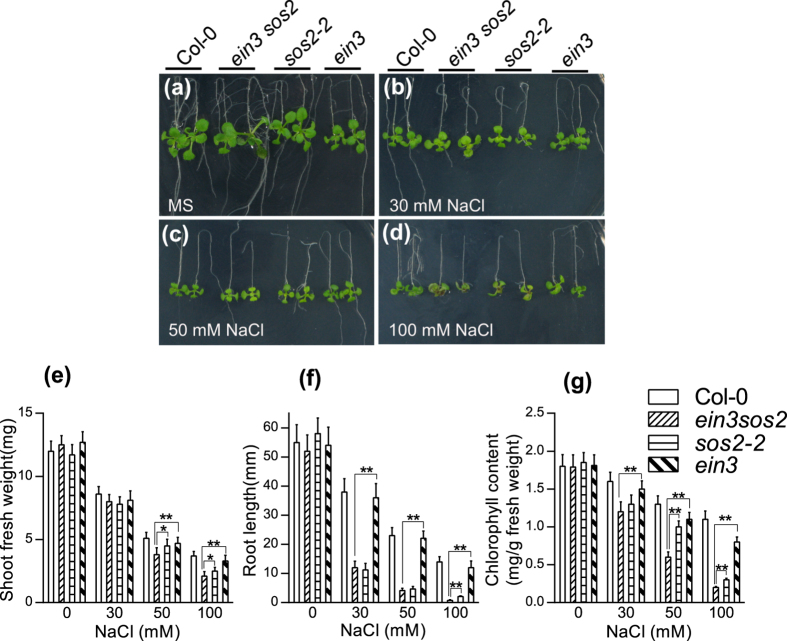
The *Arabidopsis ein3 sos2* double mutant is more sensitive to salt stress than the *ein3-1*/*sos2* single mutants at seedling stage. (**a–d**) *Arabidopsis* seedlings at 4-day stage were transferred to MS medium containing 0 (**a**), 30 (**b**), 50 (**c**) and 100 mM NaCl (**d**), respectively. (**e–g**) Shoot fresh weight (**e**), root growth (**f**) and chlorophyll content (**g**) were determined 5 days after transfer. Values are means ± SD. *Indicates significant difference at *p* = 0.05 by *t*-test; **indicates significant difference at *p* = 0.01 by *t*-test.

SOS2, a protein kinase, is required to activate the plasma membrane Na^+^/H^+^ antiporter SOS1 under salt stress^16^,^17^, and the overexpression of *SOS2* to improve kinase activity conferred increased plant salt tolerance^26^. We previously showed that the overexpression of either *EIN3* or *ESE1* to enhance the expression of salt-responsive genes improved salt tolerance in *Arabidopsis*^15^. To dissect the salt tolerance relationship between EIN3 and SOS2, we analyzed seedling growth of Col-0, *ein3-1, sos2-2* and *ein3-1 sos2* (751) in MS medium containing 0, 30, 50 and 100 mM NaCl (Fig. 1). As shown in Fig. 1a–d, the seedling growth of Col-0, *ein3-1, sos2-2* and *ein3-1 sos2* showed no difference on MS medium (Fig. 1a), but under salt stress with 30 (Fig. 1b), 50 (Fig. 1c) and 100 mM NaCl (Fig. 1d), the shoot fresh weight and root growth of *sos2-2* and *ein3-1 sos2* were less than those in Col-0 and *ein3-1* (Fig. 1e,f). Under salt stress, the leaves of *sos2-2* and *ein3-1 sos2* turned yellow-green, thus indicating less chlorophyll content. Therefore, we determined the chlorophyll content by using a spectrophotometric method after pigment extraction with 80% acetone. As shown in Fig. 1g, under salt stress, the chlorophyll content in *ein3-1 sos2* was less than that in *sos2-2.* Under 100 mM NaCl stress, the euphylla of *ein3-1 sos2* turned brown as a result of chlorophyll degradation and anthocyanin accumulation^19^.

**Figure 2:**
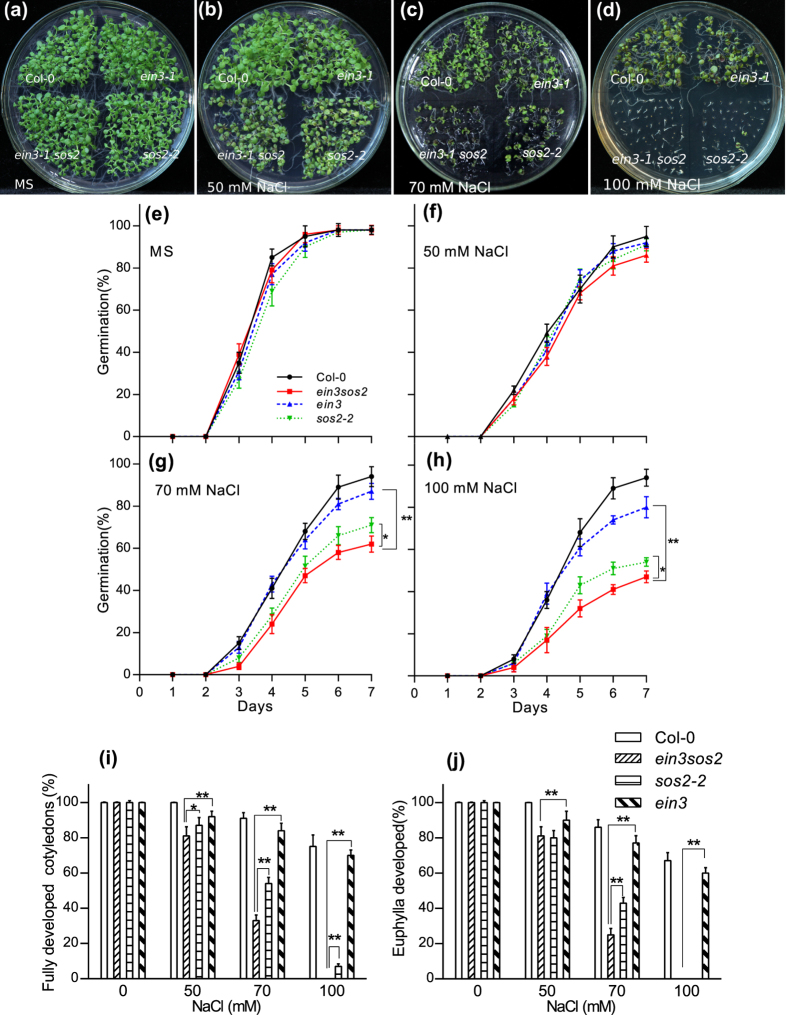
The *Arabidopsis ein3 sos2* double mutant is more sensitive to salt stress than the *ein3-1*/*sos2* single mutants during germination. (**a–h**) Germination of Col-0, *ein3-1, sos2-2*, and *ein3 sos2* double mutant seeds on MS medium containing 0 (**a,e**), 50 (**b,f**), 70 (**c,g**) and 100 mM NaCl (**d,h**). (**i**) Percentage of seedlings with fully developed cotyledons 7 days after germination. (**j**) Percentage of seedlings with euphylla 7 days after germination. Values are means ± SD. *Indicates significant difference at *p* = 0.05 by *t*-test; **indicates significant difference at *p* = 0.01 by *t*-test.

To investigate salt responses regulated by EIN3 and SOS2 in germination, we germinated Col-0, *ein3-1, sos2-2* and *ein3-1 sos2* (751) seeds in MS medium containing different concentrations of NaCl (Fig. 2). The germination rates showed no significant differences among Col-0, *ein3-1, sos2-2* and *ein3-1 sos2* seeds (Fig. 2a,e). On MS medium containing 50 mM NaCl, the germination rate of *ein3-1* was similar to that of *sos2-2* but was slower than that of Col-0, whereas the germination rate of the *ein3-1 sos2* double mutant was the lowest (Fig. 2b,f). On MS medium containing 70 or 100 mM NaCl, the germination rate of *sos2* was less than that of *ein3-1* but more than that of the *ein3-1 sos2* double mutant (Fig. 2c,d,g,h).

Seven days after germination, all seedlings on MS medium had well developed cotyledons and several euphylla, but on MS medium containing 50 mM NaCl, the euphylla of *sos2* and *ein3-1 sos2* were similar but much smaller than those of Col-0 and *ein3-1* (Fig. 2b,i,j). On MS medium containing 70 mM NaCl, the percentages of seedlings with fully developed cotyledons and euphylla of *sos2-2* were more than that of *ein3-1 sos2* but less than that of *ein3-1* (Fig. 2c,i,j). On MS medium containing 100 mM NaCl, about 80% of Col-0 developed euphylla, but almost all of *sos2* and *ein3-1 sos2* had a bleached appearance and died despite a 40% rate of germination (Fig. 2d,h,i,j).

The above results indicate that *ein3-1 sos2* double mutant is more sensitive to salt stress than *ein3-1* or *sos2-2* single mutant during the germination process and seedling growth stage. Therefore, EIN3 and SOS2 might synergistically regulate the salt stress response in *Arabidopsis*.

### EIN3 does not influence the transcription of *SOS* genes, but SOS2 positively regulates the expression of the EIN3 target gene *ESE1*

**Figure 3:**
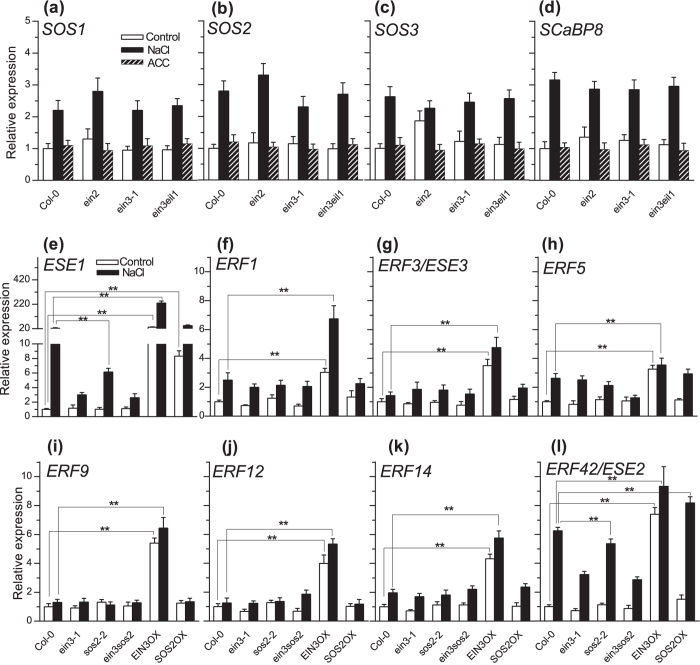
SOS2 positively regulates the expression of *ESE1*, but the expression levels of *SOS* genes are not influenced by EIN3 or EIN2. (**a–d**) The expression of *SOS* genes *SOS1* (**a**)*, SOS2* (**b**)*, SOS3* (**c**) and *SCaBP8* (**d**) in Col-0, *ein2, ein3* and *ein3 eil1* in the control, after the treatment by 150 mM NaCl for 2 hours, or by 10 μM ACC for 3 hours. (**e–l**) The expression of *ESE1* (**e**), *ERF1* (**f**), *ERF3* (**g**), *ERF5* (**h**), *ERF9* (**i**), *ERF12* (**j**), *ERF14* (**k**) and *ERF42* (**l**) in Col-0, *ein3, sos2, ein3 sos2* mutants and *EIN3/SOS2* overexpression lines before and 2 hours after treatment by 150 mM NaCl. Values are means ± SD (n = 3). **Indicates significant difference at *p* = 0.01 by *t*-test. EIN3OX and SOS2OX are *EIN3* and *SOS2* overexpression lines, respectively.

Next, we explored the regulatory relationship between EIN3 and SOS2 in the salt response. Because EIN3 is a transcription factor, we first analyzed *SOS* genes (*SOS1, SOS2, SOS3, SCaBP8*) expression in the *ein3* mutant via qPCR. As shown in Fig. 3a–d, we found that the expression of all four *SOS* genes increased 2-3 times after 150 mM NaCl treatment for 2 hours, but was not different between *ein3* and Col-0. The induction of *SOS* genes was almost similar between *ein3 eil1* double mutant and *ein3* after salt treatment. Furthermore, the expression patterns of *SOS* genes were not changed in *ein2* mutant compared with Col-0 (Fig. 3a–d). In addition, the treatment with ethylene biosynthesis precursor 1-aminocyclopropane-1-carboxylic acid (ACC) at 10 μM for 3 hours did not change the expression levels of *SOS* genes in wild type plants and ethylene signaling mutants *ein2, ein3* and *ein3 eil1* plants (Fig. 3a–d).

Previously, we identified 3 *ERF* genes that were strongly induced by both salt and ACC, and we also found 5 *ERF* genes were slightly induced by salt and ACC by examining the expression data of 122 putative *ERF* genes in Geneinvestigator databases followed by qPCR confirmation^15^. The salt and ethylene/ACC induced genes might participate in ethylene and salt responses, and therefore we determined whether SOS2 affects the expression of these putative EIN3 target *ERF* genes in the salt response (Fig. 3e–l). Under normal conditions, the basal expression level of *ESE1* was similar among Col-0, *ein3-1, sos2-2*, and *ein3 sos2.* Two hours after treatment with 150 mM NaCl, the *ESE1* induction was lower in the *ein3 sos2* double mutant than in the *ein3* or *sos2* single mutant. Constitutive expression of *SOS2* also increased the *ESE1* expression level under normal or salt stress conditions (Fig. 3e). The expression pattern of *ERF42 (ESE2*) was similar to that of *ESE1,* but induced to a lesser extent (Fig. 3l).

The expression of *ERF1, ERF3, ERF5* and *ERF14* was induced after salt stress treatment, but the expression patterns were similar in Col-0, *sos2-2*, and *SOS2* overexpression lines, whereas their expression was enhanced in *EIN3* overexpression lines (Fig. 3f–h,k). Although the transcription of *ERF9* and *ERF12* was activated by EIN3, it was not altered by SOS2 (Fig. 3e,j).

The salt induction of *ESE1* and *ERF42 (ESE2*) in *ein3 sos2* double mutant was similar to the level in *ein3-1* but less than that of *sos2*, suggesting that salt induction of ethylene-responsive genes might depend more on ethylene signaling than on SOS pathway. Therefore, SOS2 might regulate the transcription of *ERFs* by modulating ethylene signaling components.

### SOS2 phosphorylates EIN3 *in vitro*

SOS2, a Ser/Thr protein kinase in *Arabidopsis*, phosphorylates SOS1, thereby activating its Na^+^ pump activity under salt stress^17^,^26^,^27^. EIN3 is a transcription factor in the ethylene signaling pathway. *EIN3* is constitutively expressed, but EIN3 protein interacts with two F-box proteins (EBF1 and EBF2) and is degraded by the 26S proteasome without ethylene. T592 phosphorylation promotes EIN3 degradation, whereas T174 phosphorylation stabilizes EIN3, thus indicating that the stability of EIN3 is regulated by its phosphorylation status^28^.

**Figure 4:**
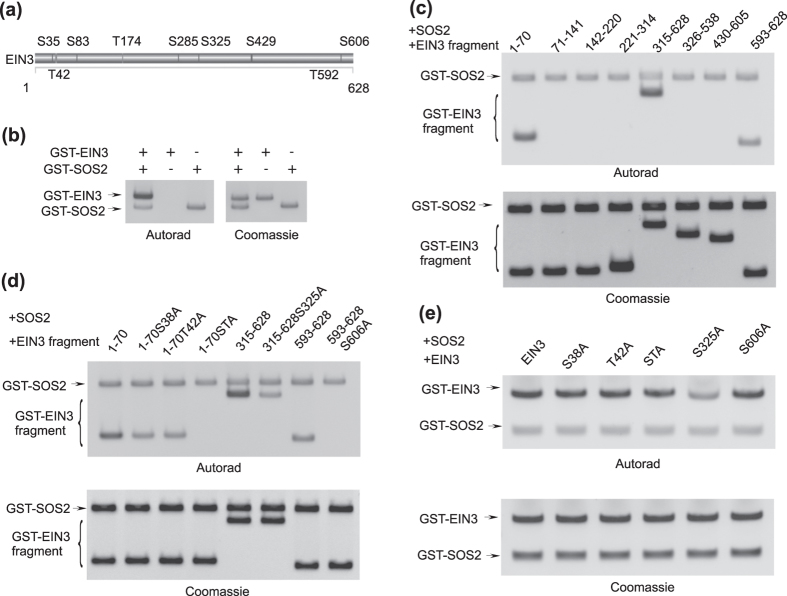
SOS2 phosphorylates EIN3 *in vitro*. (**a**) Putative sites in EIN3 phosphorylated by Ser/Thr kinase, as predicted by NetPhos (http://www.cbs.dtu.dk/ services/NetPhos/). In this prediction, nine amino acids (Ser35, Thr42, Ser83, Thr174, Ser285, Ser325, Ser429, Thr592, Ser606) are potential sites phosphorylated by Ser/Thr protein kinases. (**b**) Phosphorylation of EIN3 by protein kinase SOS2 *in vitro*. (**c–e**) Phosphorylation EIN3 fragments (**c**), EIN3 fragments with point mutations (**d**) and full length EIN3 with point mutations (**e**) by protein kinase SOS2 *in vitro*. Numbers (1–70, 71–141, 142–220, 221–314, 315–628, 326–538, 430–605, 593–628) indicate GST-EIN3 fragments with corresponding EIN3 sequences. S35A, T42A, S325A and S606A indicate mutation of Ser/Thr to Ala. STA indicates double mutation of Ser35 and Thr42 to Ala. The uncropped images are shown in Supplementary Fig. S4.

EIN3 consists of 10 putative Ser/Thr phosphorylation sites, as predicted by a bioinformatics search (Fig. 4a). To determine whether the Ser/Thr kinase SOS2 phosphorylates EIN3 and regulates downstream gene expression, we conducted an *in vitro* phosphorylation assay by incubating GST-SOS2 and GST-EIN3 recombinant proteins with γ-^32^P-ATP. As shown in Fig. 4b, full length EIN3 was phosphorylated by SOS2 *in vitro*.

To determine the sites of EIN3 phosphorylated by SOS2, we divided EIN3 into small fragments, each of which had 1–3 putative phosphorylation sites, and then conducted *in vitro* phosphorylation experiments. As shown in Fig. 4c, fragments 1–70, 315–628, and 593–628 were phosphorylated by SOS2.

Next, we analyzed the phosphorylation of EIN3 with point mutations (Fig. 4d). Fragment 1–70 consisted of two putative phosphorylation sites S35 and T42, and an *in vitro* kinase assay showed that fragment 1–70 with S35A or T42A single mutation was phosphorylated by SOS2, but the signal was much less than that of wild-type fragment 1–70. Furthermore, fragment 1–70 with the S35A T42A double mutation (1-70STA) was not phosphorylated by SOS2. Together, these results suggest that both S35 and T42 in EIN3 may be phosphorylated by SOS2 *in vitro*. Fragment 315–628 displayed the highest signal in the *in vitro* kinase assay, but fragment 315–628 with S325A mutation displayed a weak phosphorylation signal. Fragment 593–628, consisting of only one putative phosphorylation site S606, was phosphorylated, thus suggesting that S606 may be one phosphorylation site. SOS2 did not phosphorylate fragment 593–628 with the S606A mutation, thus confirming that S606 is one site in EIN3 that is phosphorylated by SOS2 *in vitro* (Fig. 4d). These results indicate that S325 might be the main phosphorylation site, and S606 might be a weak phosphorylation site. In addition, phosporylation analysis of full length EIN3 with point mutations by SOS2 revealed that S325A greatly reduced EIN3 phosphorylation, and S35A, T42A or S606A only slightly reduced EIN3 phosphorylation (Fig. 3e). Therefore, S325 may be the main phosphorylation site, whereas S35, T42 and S606 might be weak sites in EIN3 that are phosphorylated by SOS2 *in vitro*.

In *Arabidopsis*, EIN3 has 5 homologs, EIL1~EIL5. To determine whether the phosphorylation sites of EIN3 are conserved among EIN3/EIL proteins, we performed multiple sequence alignments using CLUSTAL 2.0.12^29^ (Supplementary Fig. S3). Among the four phosphorylation sites of EIN3, S325 is the most conserved amino acid, followed by T42, whereas the positions of S35 and S606 are highly diverse. Because S325, the main EIN3 site phosphorylated by SOS2 *in vitro* is conserved among the EIN3/EIL family, S325 might be crucial for the function of EIN3/EILs.

### EIN3 S325A mutation reduces its transcriptional activating activity on *ESE1,* and impairs its ability to rescue *ein3-1* salt hypersensitivity

**Figure 5:**
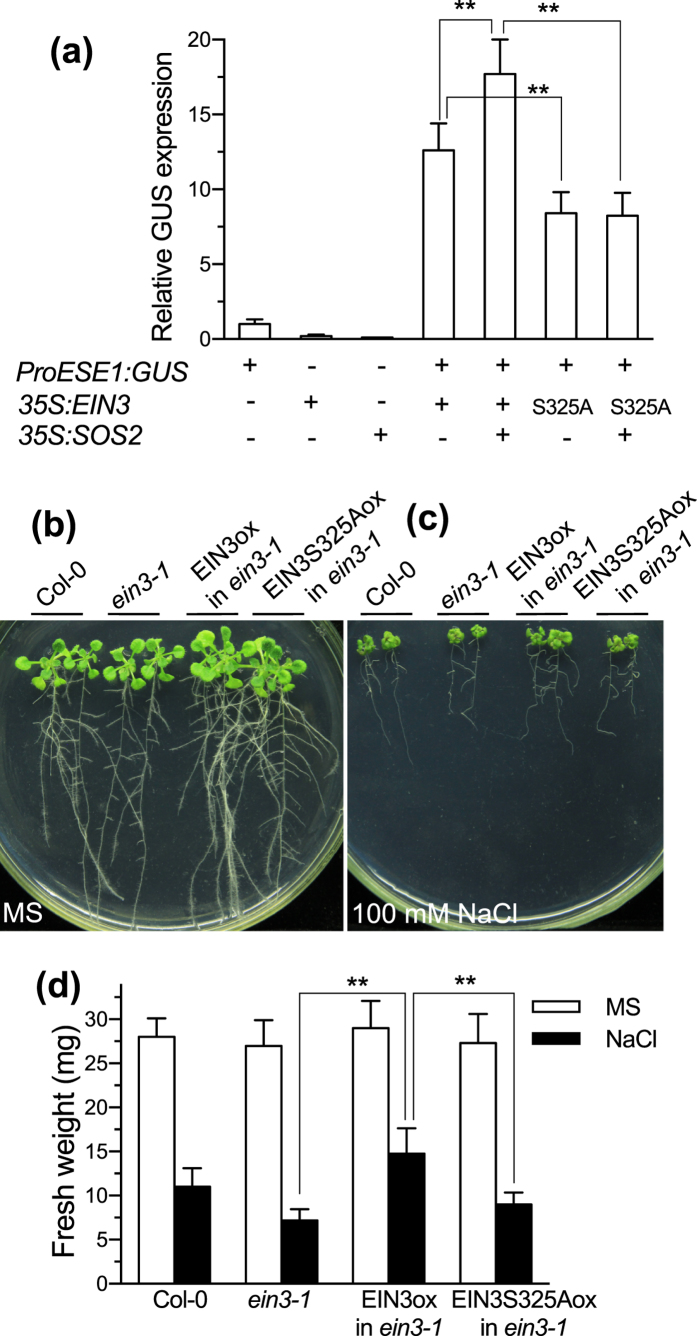
EIN3 S325A mutation reduces its transcriptional activation on *ESE1* and impairs its ability to rescue *ein3–1* salt hypersensitivity. (**a**) Transient GUS assay for *ESE1* promoter transcriptional activity by EIN3 and its mutated protein in tobacco leaves. 35S:LUC is an internal control. Values are means ± SD (n = 5). **Indicates significant difference at *p* = 0.01 by *t*-test. (**b–d**) Seedling at 4-day stage were transferred to MS medium with 0 (**a**) and 100 mM NaCl. Ten days after transfer, photos were captured (**b,c**) and shoot fresh weight per plant was determined (**d**). Pictures are representative photos of 3 independent transgenic lines with similar results. Values are means ± SD with 3 independent transgenic lines in 3 repetitions. **Indicates significant difference at *p* = 0.01 by *t*-test.

To determine the effect of S325 phosphorylation in EIN3 to its transcriptional activating activity on *ESE1*, we performed a transient assay by co-transformation of 35S:EIN3, 35S:SOS2 and the reporter ESE1 promoter:GUS with 35S:LUC as an internal control (Fig. 5a). The results showed that the transcriptional activity of *ESE1* promoter was reduced at about 30% by S325A mutation in EIN3. Furthermore, SOS2 increased the transcriptional activating activity of EIN3, but not EIN3S325A, on *ESE1* promoter at about 25%. These results suggest that S325 phosphorylation is critical for the transcriptional activity of *ESE1* regulated by EIN3 and SOS2.

Next, we investigated the salt tolerance of *EIN3* or *EIN3S325A* overexpression lines in *ein3-1* background (Fig. 5b–d). The results showed that the overexpression of EIN3 improved *ein3-1* salt tolerance in root growth and shoot fresh weight, whereas the overexpression of EIN3S325A only partially rescued *ein3-1* salt hypersensitivity.

The above results indicate that the phosphorylation of EIN3S325 by SOS2 is critical for ESE1 transcriptional activation *in vivo* and plant salt tolerance, at least partially.

### EIN3 and SOS2 coactivate downstream salt-responsive gene expression

**Figure 6:**
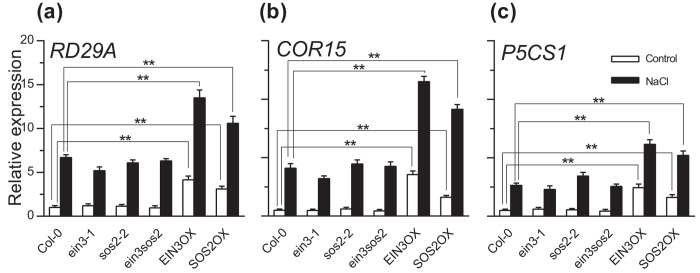
EIN3 and SOS2 activate stress-responsive genes under salt stress. (**a–c**) The expression of *RD29A* (**a**), *COR15* (**b**) and *P5CS1* (**c**) in Col-0, *ein3, sos2, ein3 sos2* mutants and *EIN3/SOS2* overexpression lines before and 2 hours after treatment by 150 mM NaCl. Values are means ± SD (n = 3). **Indicates significant difference at *p* = 0.01 by *t*-test. EIN3OX and SOS2OX are *EIN3* and *SOS2* overexpression lines, respectively.

As shown above, SOS2 activated the expression of the EIN3 target gene *ESE1*, and SOS2 phosphorylated EIN3 *in vitro*; therefore, the phosphorylation of EIN3 by SOS2 under salt stress might upregulate *ESE1* expression. Previously, we have found that *ese1* is salt sensitive (Supplementary Fig. S5)^15^, and EIN3 and ESE1 activate the expression of salt-responsive genes, including *RD29A*^30^, *COR15*^31^ and *P5CS*^32^, by binding to their promoter regions and activating gene expression^15^. Previous studies showed that the loss-of-function mutant of *p5cs1* is salt sensitive^32^. In salt tolerance test, seedling growth was inhibited much more by salt stress in *rd19a* and *p5cs1* than in wild type plants, but the growth of *cor15* was almost similar to wild type plants in salt stress (Supplementary Fig. 6a–f). To investigate whether EIN3 and SOS2 coactivated salt-responsive gene expression, we analyzed the expression of *RD29A, COR15* and *P5CS1* in *ein3-1, sos2-2, ein3 sos2*, EIN3OX and SOS2OX in control and salt-stressed plants (Fig. 6a–c). Under normal conditions, the expression of *RD29A, COR15* and *P5CS1* was not significantly different in *ein3-1, sos2-2, ein3 sos2* mutants and wild type but was upregulated in EIN3OX and SOS2OX plants. After salt stress, the expression of *RD29A, COR15* and *P5CS1* was induced by salt treatment in all plants, but the expression was much higher in EIN3OX and SOS2OX than in the other plants. These results suggest that both EIN3 and SOS2 positively regulate salt-responsive gene expression.

## Discussion

Both ethylene biosynthesis^8^ and the ethylene signaling^4^,^13^,^14^ play positive roles in *Arabidopsis* salt tolerance. In *Arabidopsis,* the SOS pathway, consisting of SOS1^16^, SOS2^17^, SOS3^18^ and SCaBP8^19^, is crucial for plant salt tolerance by regulating iron homeostasis under conditions of salt stress. In this study, we isolated two new *sos2* alleles as salt sensitive mutants in the *ein3-1* background, and found SOS2 phosphorylated and activated EIN3, which suggests a connection between the ethylene signaling component EIN3 and the SOS pathway component protein kinase SOS2 in plant salt response regulation.

EIN3 is a transcription factor, but neither EIN2 nor EIN3 changes the expression pattern of *SOS* genes under normal or salt stress conditions. However, SOS2 activates the expression of *ESE1*, which encodes an ERF transcription factor downstream of EIN3. Microarray analysis in *sos2* mutant has demonstrated that some *EREBP* genes and *ACS* gene are upregulated^25^, thus implying a crosstalk between the SOS pathway and ethylene signaling in plant salt responses. *In vitro* phosphorylation of EIN3 by SOS2 and the consequent upregulation of the *ERF* gene downstream of EIN3 indicate that SOS2-EIN3 might serve as junction between the SOS pathway and the ethylene signaling pathway in plant salt response regulation.

Protein phosphorylation is one means of regulation of EIN3 in ethylene signaling^28^. T174 and T592 are two phosphorylation sites of EIN3 that affect EIN3 stability in opposite ways. Without ethylene, CTR1, a Raf-like MAPK kinase kinase, inactivates MKK9–MPK3/6 and activates downstream MAPKs, which in turn phosphorylate T592 and promote EIN3 degradation. Ethylene inactivates CTR1, thus resulting in MKK9-MPK3/6 activation and T174 phosphorylation and the consequent stabilization of EIN3. In this study, we found that SOS2 phosphorylates S325, but not T174 or T592 of EIN3 in kinase assays *in vitro*. These results suggest that phosphorylation of EIN3 at different positions might have various functions: T174 and T592 may act in ethylene signaling, and S325 in salt responses.

Previous studies have shown that the expression of many stress-responsive genes changes in *sos* mutants^16^,^25^,^33^. However, it is unknown how protein kinase SOS2 regulates gene expression. In this study, we found that the mutation of EIN3 S325A, a phosphorylation site by SOS2, reduces its transcriptional activating activity on *ESE1* promoter:GUS, and weakens its ability to rescue *ein3-1* salt hypersensitivity, revealing that SOS2 might regulate plant salt response by phosphorylating EIN3 to transcriptionally activate downstream salt stress responsive genes. Therefore, EIN3 and SOS2 might be linked together to modulate plant salt stress response via the phosphorylation of EIN3 by SOS2.

Salt stress increases EIN3 protein accumulation by promoting the proteasomal degradation of EBF1 and EBF2^34^. Meanwhile, salt stress induces cytosolic calcium accumulation, which is sensed by the calcium binding proteins SOS3 and SCaBP8^18^,^19^,^20^. Then SOS2, which is activated by SOS3 or SCaBP8, phosphorylates and activates SOS1^1^,^16^,^17^,^18^,^19^,^20^,^21^,^22^. In this study, we found SOS2 phosphorylates EIN3 protein, which expands the range of proteins phosphorylated by SOS2 in addition to SOS1 and SCaBP8^20^,^21^. Thus the phosphorylation of EIN3 by SOS2 might increase EIN3 protein stability or directly enhance EIN3 transcriptional activity. Therefore, EIN3 and SOS2 synergistically modulate plant salt tolerance.

Since either the overexpression of *EIN3*^15^ or the overexpression of *SOS2* to improve kinase activity^26^ increases plant salt tolerance, and the expression of *SOS* genes is not changed in *ein3* and *ein2* mutants or by ACC treatment, future experiments to test the salt tolerance of *EIN3* overexpresser in *sos2* background and *SOS2* overexpresser in *ein3* background would provide more cues for the genetic interaction between SOS2 and EIN3 in plant salt response regulation. In addition, the function of the other putative sites in EIN3 phosphorylated by SOS2 in salt response regulation needs to be addressed in future investigations. And whether the phosphorylation of EIN3 by SOS2 is regulated by other factors is unknown yet. Furthermore, to dissect additional components connecting the SOS and ethylene pathways is an interesting topic.

In summary, through genetic screening, we identified *sos2* as a salt sensitive mutant in the *ein3-1* background. SOS2 phosphorylates EIN3 and consequently activates downstream *ERF* gene expression and induces stress responsive gene expression under salt stress conditions. Therefore, SOS2-EIN3 might link the SOS and ethylene pathways in plant salt responses.

## Methods

### Isolation of salt sensitive mutants in an *ein3-1* background

Approximately 20,000 *ein3-1* seeds were treated with 25 volumes of 0.2% (v/v) ethyl methane sulfonate (EMS) for 15 hours. The seeds were washed 10 times with water after removal of the EMS. Seeds were suspended in 0.1% agarose and sown on soil. M2 seeds were collected in pools after plants had matured^35^.

M2 seeds of *ein3-1* were sterilized in a solution containing 20% sodium hypochlorite and 0.1% Triton X-100 for 10 min, washed five times with sterilized water, and sown on MS medium with 0.6% Phytagel (Sigma-Aldrich, P8169). The plates were placed at 4 °C for 2 days, and then the seeds were germinated vertically at 23 °C under 16/8 (light/dark) hours illumination. Four-day-old seedlings with a root length of 1.5 cm were transferred onto MS medium supplemented with 100 mM NaCl and cultured vertically with the roots upward and shoots downward. The root growth and shoot growth was checked after cultivation for 4 days^19^.

Seedlings with a hyper salt sensitive phenotype were planted in soil to collect M3 seeds. After confirmation of the salt sensitive phenotype in M3 seedlings, M3 plants were crossed with Ler-0. Then, the mutation sites were determined by map-based cloning using F2 segregating population.

### Salt sensitivity analysis in *Arabidopsis*

The salt sensitivity analysis of *Arabidopsis* seedlings was carried out as described above. For salt sensitivity analysis of *Arabidopsis* in germination, the seeds were sterilized in a solution containing 20% sodium hypochlorite and 0.1% Triton X-100 for 10 min, washed five times with sterilized water, and sown on MS medium (0.2% Phytagel, Sigma-Aldrich, P8169) with 0, 50, 70 or 100 mM NaCl added. The plates were incubated at 4 °C for 2 days, and the seeds were germinated at 23 °C under 16/8 (light/dark) hours illumination. The germination rate was measured daily.

### Chlorophyll content determination

*Arabidopsis* leaves were weighed and fractured in 80% acetone in water to extract chlorophyll. The debris was removed by centrifugation, and the absorbance of the supernatant at 646 and 663 nm was determined with a spectrometer; the chlorophyll concentration was calculated as described previously^36^.

### Gene expression analysis via real-time quantitative PCR (qPCR)

For *Arabidopsis*, 7-day seedlings were treated by 150 mM NaCl for 2 hours, and leaves were collected and stored in liquid nitrogen. For rice, 2-week seedlings were stressed with 100 mM NaCl for 2 hours or 150 mM NaCl for 3 hours, and leaves were collected and stored in liquid nitrogen. Total RNA from leaves was extracted via the TRIzol method (ThermoFisher)^37^,^38^ and digested with DNase free RNase to remove genomic DNA. Reverse transcription was performed using 1 mg of total RNA and MLV reverse transcriptase (Promega). The cDNA was then used for PCR amplification with gene specific primers in Table S1 on a real-time PCR machine (Bio-rad IQ5). The gene expression level was calculated with Bio-rad IQ5 software (version 2.1).

### Recombinant proteins expression and purification

Recombinant SOS2 and EIN3 proteins were expressed as GST fusions. GST-SOS2 has been described previously^19^,^20^. For GST-EIN3, an EIN3 full-length coding region was amplified using primers with *Bam*HI and *Sal*I sites (Table S1) from *Arabidopsis* cDNA. The PCR product was digested with *Bam*HI and *Sal*I and cloned into a pGEX-6P-1 vector. For GST-EIN3 fragments, EIN3 fragment coding region was amplified with the corresponding primers in Table S1 by using a GST-EIN3 construct as a template, digested with *Bam*HI and *Sal*I, and cloned into the pGEX-6P-1 vector (GE Healthcare 28-9546-48). To generate EIN3 with a Ser/Thr to Ala point mutation, we introduced the mutation sites by PCR *in vitro* via site-directed mutagenesis using primers in Table S1^39^. The EIN3 S35A, T42A double mutant was generated by introducing T42A in EIN3 S35A through the method described above.

The GST fusion constructs were transformed into *Escherichia coli* strain BL21 (DE3). The expression of recombinant proteins was induced with 0.5 mM IPTG at 18 °C for 6 hours. The cells were then collected by centrifugation and lysed by sonication. The recombinant proteins were purified with glutathione Sepharose 4B (GE Healthcare 17075601) according to the manufacturer’s protocol.

### *In vitro* kinase assays

*In vitro* kinase assays were performed in kinase buffer including 20 mM Tris-HCl, pH 8.0, 5 mM MgCl_2_, 10 μM ATP, and 1 mM DTT. In a total volume of 20 μL, 1 μg protein and 0.5 μL of [γ-^32^P] ATP (5 μCi) were added to the kinase buffer and incubated at 30 °C for 30 min. The reactions were terminated by the addition of 4 μL 6 × SDS loading buffer, then incubated at 95 °C for 5 min. The proteins were separated via 12% (w/v) SDS-PAGE and stained with Coomassie brilliant blue R250, and then the signals were captured with an X-film (Kodak) or a phosphor screen (Amersham Biosciences)^19^,^20^.

### Sequence alignments

Protein sequences of EIN3 and EIL1~5 were downloaded from The Arabidopsis Information Resource (TAIR) (http://www.arabidopsis.org/), and multiple sequence alignments were performed with CLUSTAL 2.0.12^29^. The alignment output was printed out with BoxShade (http://www.ch.embnet.org/software/BOX%5Fform. html).

### Transient GUS assay

A transient GUS assay was performed by transient expression in tobacco leaves as described previously^15^,^40^. The constructs ProESE1:GUS, 35S:EIN3(/S325A), 35S:SOS2 and 35S:LUC were transferred into *Agrobacterium* strain GV3101 separately by electroporation. Agrobacteria were harvested and incubated with induction medium containing 100 μM acetosyringone. Then the bacteria were injected into tobacco leaves that still attached to the intact plant. Two days after agroinfiltration, GUS activity was determined using 4-methylumbelliferyl-D-glucuronide as a substrate, and luciferase activity was used as an internal control.

## Additional Information

**How to cite this article:** Quan, R. *et al*. EIN3 and SOS2 synergistically modulate plant salt tolerance. *Sci. Rep.* **7**, 44637; doi: 10.1038/srep44637 (2017).

**Publisher's note:** Springer Nature remains neutral with regard to jurisdictional claims in published maps and institutional affiliations.

## Supplementary Material

Supplementary Information
