# Supplementary material for: EIN3 and SOS2 synergistically modulate plant salt tolerance
Source: Sci Rep. 2017 Mar 16;7:44637. doi: 10.1038/srep44637 (PMC5353744; doi:10.1038/srep44637)
Supplement: Supplementary Information [file srep44637-s1.pdf]

Supplementary Information

EIN3 and SOS2 synergistically modulate plant salt tolerance

Ruidang Quan[1, 2], Juan Wang[1, 2], Dexin Yang[1, 2], Haiwen Zhang[1, 2], Zhijin Zhang[1, 2] & Rongfeng Huang[1, 2,*]

[1]Biotechnology Research Institute, Chinese Academy of Agricultural Sciences, 12 Zhongguancun South Road, Beijing 100081, China.

[2]National Key Facility of Crop Gene Resources and Genetic Improvement, 12 Zhongguancun South Road, Beijing 100081, China

Correspondence and requests for materials should be addressed to R.H. (email: rfhuang@caas.cn)

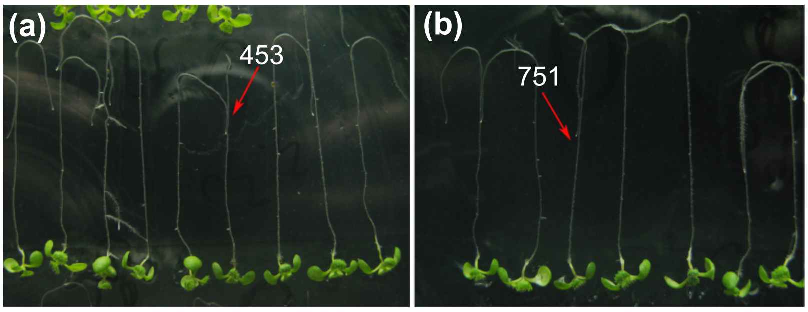

100 mM NaCl

Supplementary Figure S1. Screening for salt sensitive mutants in *ein3-1* background.

(a, b) M2 seeds of EMS-mutagenized *ein3-1* were germinated on MS media and 4-day seedlings were transferred to MS media containing 100 mM NaCl. Salt sensitive mutants were characterized by growth retardation in shoots and/or roots of *ein3-1* seedlings. 453 (a) and 751 (b) were two putative salt sensitive mutants in *ein3-1* background.

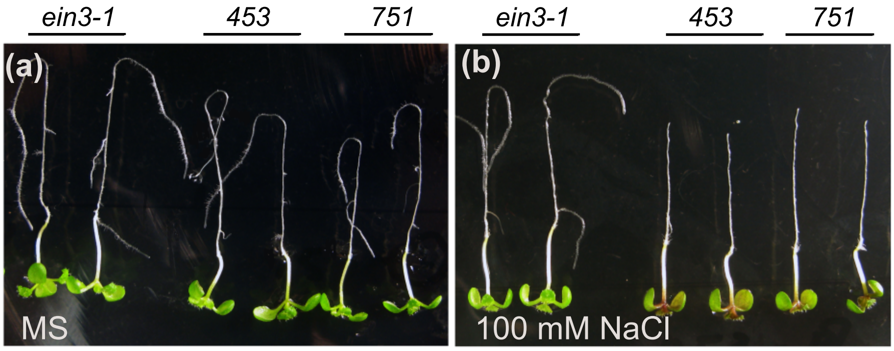

Supplementary Figure S2. Both mutants 453 and 751 are more sensitive to salt stress than *ein3-1*.

Seeds were germinated on MS media and 4-day seedlings were transferred to MS media containing 0 (a) and 100 mM NaCl (b), and photos were taken after cultivation for 4 days.

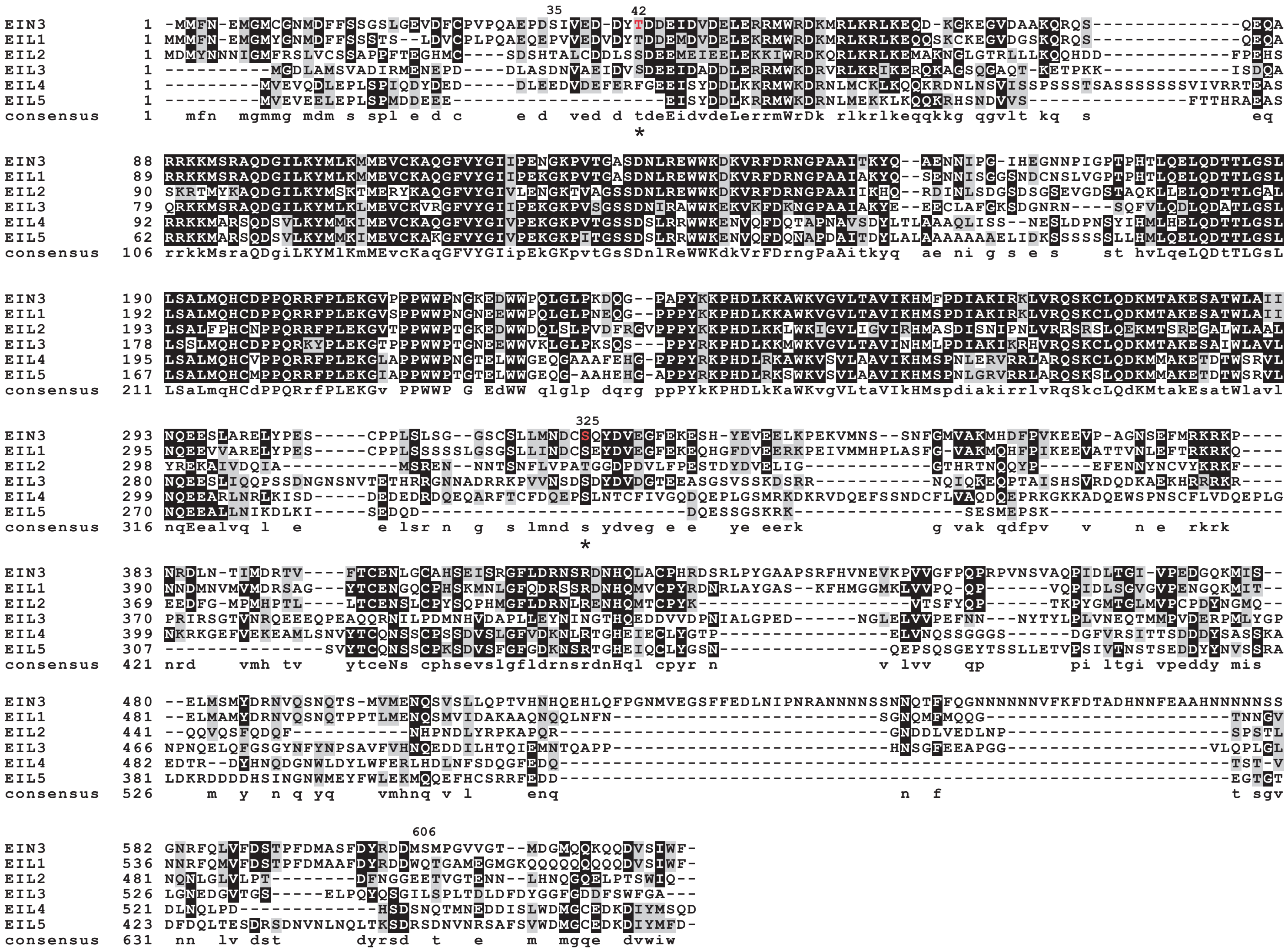

Supplementary Figure S3. Sequence alignments of EIN3/EIL proteins in *Arabidopsis*.

EIN3/EIL protein sequences were aligned by CLUSTAL 2.0.12 and the output was printed out with BoxShade (http://www.ch.embnet.org/software/BOX%5Fform.html). EIN3 phosphorylation sites were marked with the amino acid position numbers, and the conserved EIN3 phosphorylation sites by SOS2 in vitro are marked with stars below.

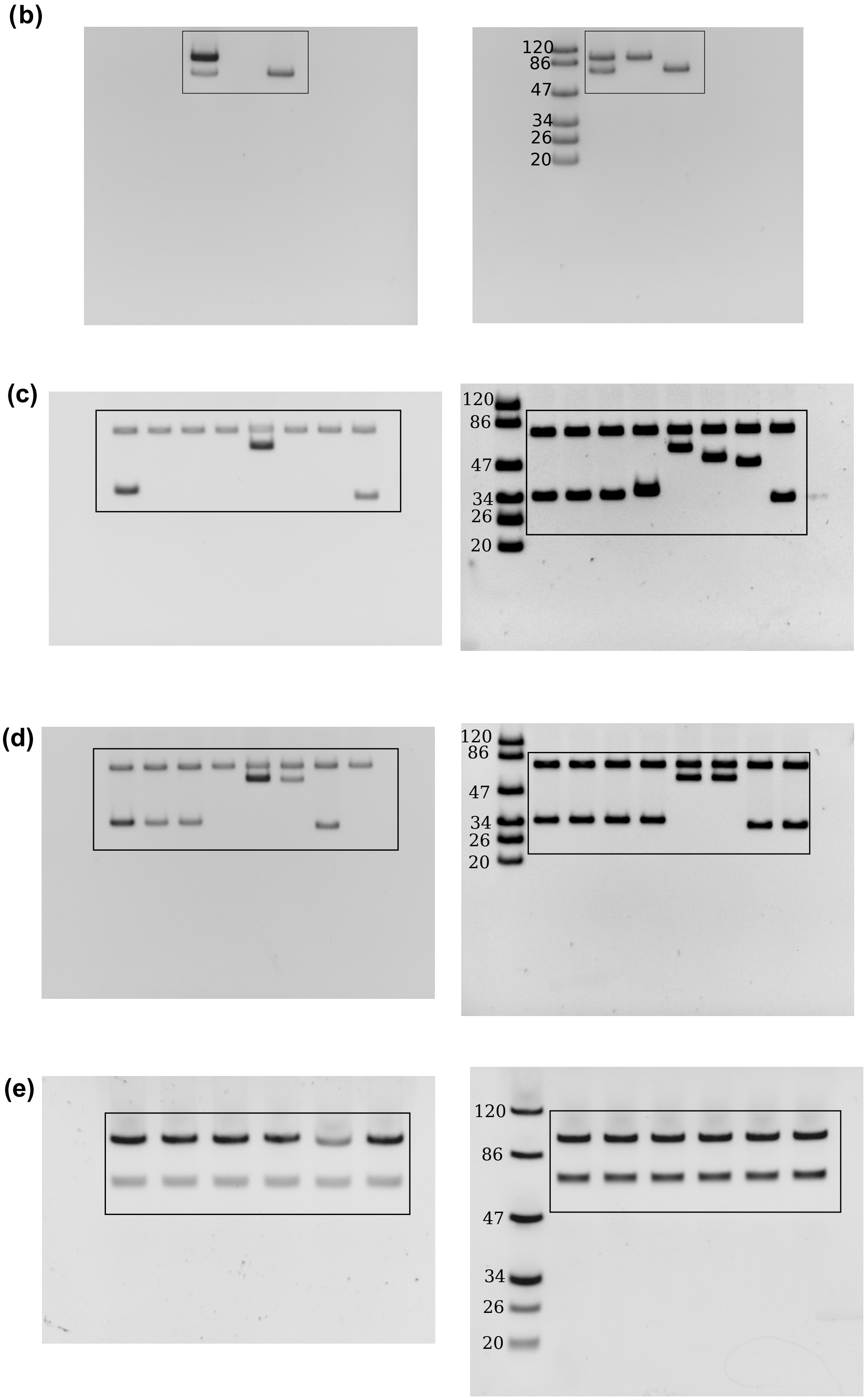

Supplementary Figure S4. Uncropped images of Figure 4.

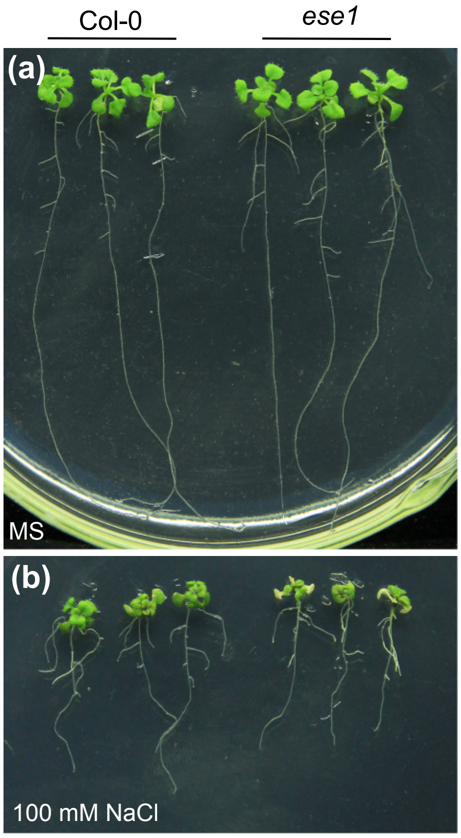

Supplementary Figure S5. *ese1* is salt sensitive than wild type plants.

Seedling at 4-day stage were transferred to MS medium with 0 (a) and 100 mM NaCl (b). Ten days after transfer, photos were captured.

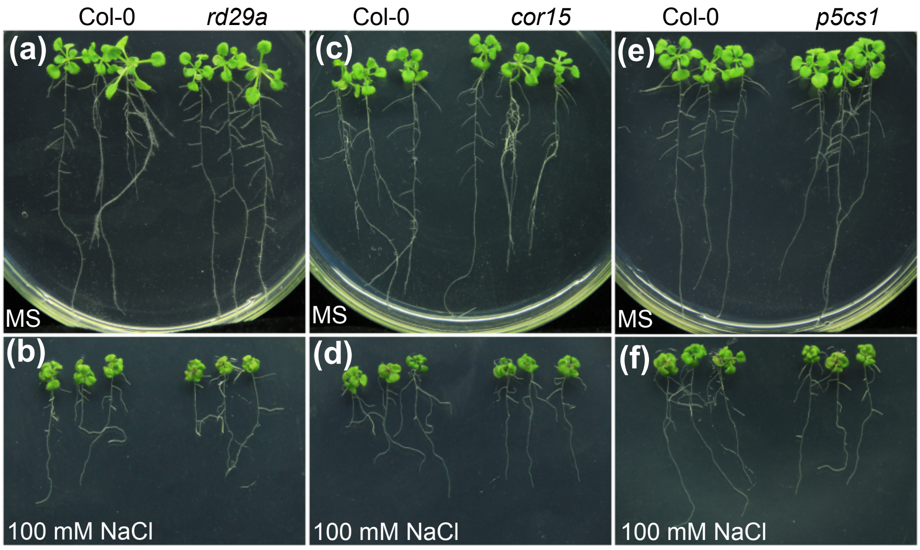

Supplementary Figure S6. Salt tolerance analysis of *rd29a*, *cor15* and *p5cs1*.

Seedling at 4-day stage were transferred to MS medium with 0 (a, c, e) and 100 mM NaCl (b, d, f). Ten days after transfer, photos were captured.

Supplementary Table S1. Primers used in this study.

Name	Sequence (5' to 3')	
ACT2RTF	GTCGTACAACCGGTATTGTG	qPCR, internal control
ACT2RTR	GAGCTGGTCTTTGAGGTTTC	qPCR, internal control
UBQ10RTF	ACCGGAAAGACTATCACTTTG	qPCR, internal control
UBQ10RTR	AGAGATAACAGGAACGGAAAC	qPCR, internal control
SOS1RTF	CCGCATTATCACCTTCTAAC	qPCR
SOS1RTR	AGATGGTCTTCGATGTAGAG	qPCR
SOS2RTF	CGAAACTTCAAGACAAGGCTC	qPCR
SOS2RTR	GTGCCACCTCGTAAATCTCTATC	qPCR
SOS3RTF	GTAATGGTGGATAAGGCTTTCG	qPCR
SOS3RTR	TGAGCGATGGATTCAAGGATAC	qPCR
SCaBP8RTF	AACAAATGGTTTCTGCTATTCTC	qPCR
SCaBP8RTR	ATCTGCATCAGCAAATGTTTTATC	qPCR
ESE1RTF	GGGACGTTTAACACAGCGGAAGAC	qPCR
ESE1RTR	GCATTCTCGTGGCTGCCATTTG	qPCR
ESE2RTF	GAAGCCGCTCTTATGGATTTC	qPCR
ESE2RTR	TCGGCAACTCCACAATATCTC	qPCR
ERF1RTF	CGGATCTTCTCCAGATTCTTTC	qPCR
ERF1RTR	CTCTGACTTTCTTGAGCTTACG	qPCR
ERF3RTF	GCCATTCCTACTTCCTCTTC	qPCR
ERF3RTR	TCTCCGTTACTCCTCTGTTC	qPCR

Supplementary Table S1. Primers used in this study.

Name	Sequence (5' to 3')	
ERF5RTF	GAAAGTGTTGAAGACGGAACAG	qPCR
ERF5RTR	GAATAACCAAACGGTGGATGAG	qPCR
ERF9RTF	ATGTCACCGGGAGCGTAAAG	qPCR
ERF9RTR	CAAACCGAACCGGACAAACC	qPCR
ERF12RTF	TACTTCATCAGCCGTCTCGTCAC	qPCR
ERF12RTR	CTCCAAAGCGGAATGTCAGCATC	qPCR
ERF14RTF	AAGTCTCCTTGTCTGAATC	qPCR
ERF14RTR	CAGCAGAAGTTCCATAATC	qPCR
ERF42RTF	GAAGCCGCTCTTATGGATTTC	qPCR
ERF42RTR	TCGGCAACTCCACAATATCTC	qPCR
EIN2CRTF	ACTGTGGAGATGGTTGCGTTTG	qPCR
EIN2CRTR	AAAGCACGGTGTCATTGGTGTC	qPCR
EIN3RTF	TAACAGTAGCGGCAACAGGTTC	qPCR
EIN3RTR	TCTTGCTGCTTCTGCTGCATTC	qPCR
GST-EIN3F	CGGGATCCATGATGTTTAATGAGATGGG	GST fusion protein
GST-EIN3R	GCGTCGACTTAGAACCATATGGATACATC	GST fusion protein
EIN3-1-70F	CGGGATCCATGATGTTTAATGAGATGGG	EIN3 fragment
EIN3-1-70R	GCGTCGACCTACTTATCCTGCTCCTTGAGAC	EIN3 fragment
EIN3-71-141F	CGGGATCCGGTAAAGAAGGTGTTGATGC	EIN3 fragment
EIN3-71-141R	GCGTCGACCTACTTATCTTTCCACCACTCCC	EIN3 fragment

Supplementary Table S1. Primers used in this study.

Name	Sequence (5' to 3')	
EIN3-142-220F	CGGGATCCGTTAGGTTTGATCGTAATGG	EIN3 fragment
EIN3-142-220R	GCGTCGACCTACTCTTTCCCATTAGGCCACC	EIN3 fragment
EIN3-221-314F	CGGGATCCGATTGGTGGCCTCAACTTGG	EIN3 fragment
EIN3-221-314R	GCGTCGACCTAACCAGACAGAGAAAGAGGTG	EIN3 fragment
EIN3-315-628F	CGGGATCCGGAAGTTGCTCGCTTCTGATG	EIN3 fragment
EIN3-315-628R	GCGTCGACTTAGAACCATATGGATACATC	EIN3 fragment
EIN3-326-538F	CGGGATCCCAATACGATGTTGAAGGTTTC	EIN3 fragment
EIN3-326-538R	GCGTCGACCTATGCTCTGTTTGGGATGTTCAAG	EIN3 fragment
EIN3-430-605F	CGGGATCCCGCTTACCGTATGGAGCAGC	EIN3 fragment
EIN3-430-605R	GCGTCGACTCACATATCATCTCTGTAATCGAATG	EIN3 fragment
EIN3-593-628F	CGGGATCCCCGTTCGACATGGCGTCATTC	EIN3 fragment
EIN3-593-628R	GCGTCGACTTAGAACCATATGGATACATC	EIN3 fragment
EIN3-S606AF	TTACAGAGATGATATGGCGATGCCAGGAGTAG	EIN3 point mutation
EIN3-S606AR	CTACTCCTGGCATCGCCATATCATCTCTGTAA	EIN3 point mutation
EIN3-S35AF	TCCACAAGCTGAGCCTGATGCCATTGTTGAAGATG	EIN3 point mutation
EIN3-S35AR	CATCTTCAACAATGGCATCAGGCTCAGCTTGTGG	EIN3 point mutation
EIN3-T42AF	GAAGATGACTATGCTGATGATGAGATTGATGTTG	EIN3 point mutation
EIN3-T42AR	CAATCTCATCATCAGCATAGTCATCTTCAACAATGG	EIN3 point mutation
EIN3-S325AF	TGATGAATGATTGCGCTCAATACGATGTTGAA	EIN3 point mutation
EIN3-S325AR	TTCAACATCGTATTGAGCGCAATCATTCATCA	EIN3 point mutation
